# Incidental Identification of a Persistent Left Superior Vena Cava During Central Venous Catheterization: A Case Report

**DOI:** 10.7759/cureus.88847

**Published:** 2025-07-27

**Authors:** Uzain Sardar, Hamza Ali Bukhari, Anas J Khan, Qasim Zia, Hasan Raza, Muhammad Moazzam, Shahzad Ahmad

**Affiliations:** 1 Anesthesia and Critical Care, Buch International Hospital, Multan, PAK; 2 Anesthesiology and Critical Care, Buch International Hospital, Multan, PAK; 3 Medicine, Ibne Siena Hospital, Multan Medical &amp; Dental College (MMDC), Multan, PAK; 4 Anesthesiology, Buch International Hospital, Multan, PAK; 5 Anesthesia and Critical Care, Multan Institute of Kidney Diseases, Multan, PAK; 6 Critical Care Medicine, Shifa International Hospitals Limited, Islamabad, PAK; 7 Surgery, Liaquat National Hospital, Karachi, PAK

**Keywords:** anatomical vascular variant, catheter malposition, central venous catheterization, incidental finding, persistent left superior vena cava (plsvc), superior vena cava anomaly

## Abstract

A rare vascular condition called persistent left superior vena cava (PLSVC) is frequently found incidentally when performing central venous catheterization (CVC). We report the case of a 57-year-old man, whose left-sided central line seemed to be incorrectly positioned on a chest X-ray with no associated anomalies; further imaging confirmed the presence of a PLSVC, which drained into the coronary sinus. It is important to recognize this variant in order to prevent diagnostic confusion and complications. PLSVC is typically asymptomatic, but PLSVC poses risks during invasive cardiac treatments. For safe clinical practice, it is crucial to be aware of this anatomical variation.

## Introduction

Central venous catheterization (CVC), also called a central line, is a thin, flexible, and large tube that is inserted into the central large veins to provide secure and long-term vascular access [1]. The indication for CVC is for medication administration, hemodynamic monitoring, total parenteral nutrition, dialysis, plasmapheresis, and post-cardiac bypass surgery and to facilitate further procedures such as pacemaker placement [2]. It is medically important to ensure safety precautions during CVC to preferred sites include the internal jugular vein, subclavian vein, or femoral vein, with the catheter tip ideally positioned in the superior vena cava (SVC) near its junction with the right atrium [3]. The SVC is a large, central vein that drains deoxygenated blood from the upper body, head, neck, arms, and upper chest into the right atrium of the heart. SVC is formed during embryological development from the right anterior cardinal vein, which persists and matures into the right SVC [4]. During fetal development, both right and left anterior cardinal veins are present. Later, the left anterior cardinal vein regresses, and the right develops into the mature SVC. However, in some individuals, the left anterior cardinal vein fails to regress and causes persistent left superior vena cava (PLSVC). The incidence of PLSVC is between 0.3% and 0.5% and up to 4.3% in patients with congenital heart diseases [5]. PLSVC coexists with a right-sided SVC and drains into the right atrium via the coronary sinus. PLSVC has clinical consequences when it drains into the left atrium, including right-to-left shunt [6]. Failure to recognize PLSVC may result in catheter malposition, arrhythmias, or diagnostic confusion on imaging studies. In this case report, we describe the incidental identification of a PLSVC during routine central venous catheter insertion in an adult patient. We aim to highlight the importance of recognizing this anatomical variant to avoid complications and ensure proper catheter positioning.

## Case presentation

**Figure 1:**
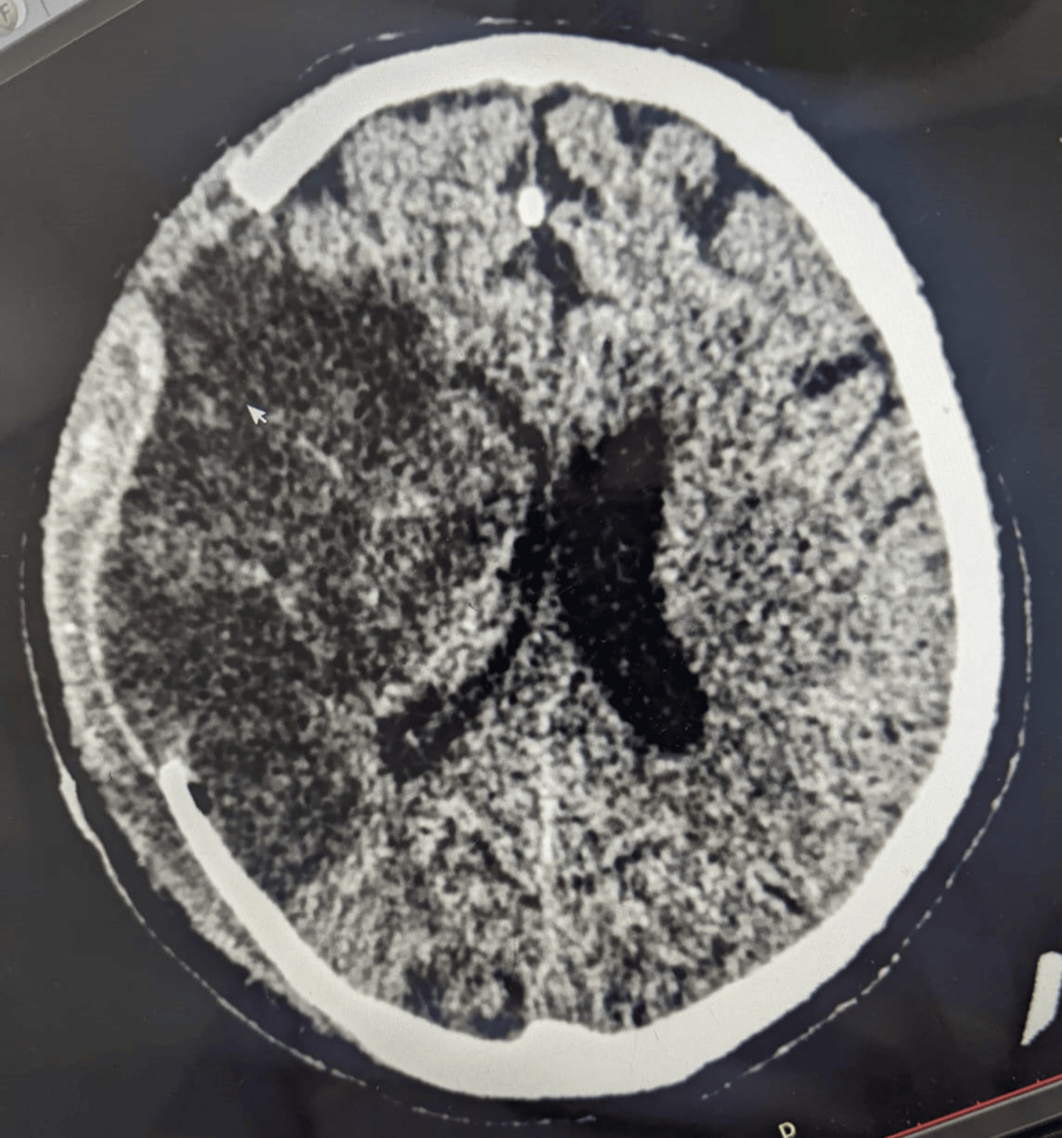
Non-contrast CT scan of the head revealed an infarct in the right middle cerebral artery territory.

**Figure 2:**
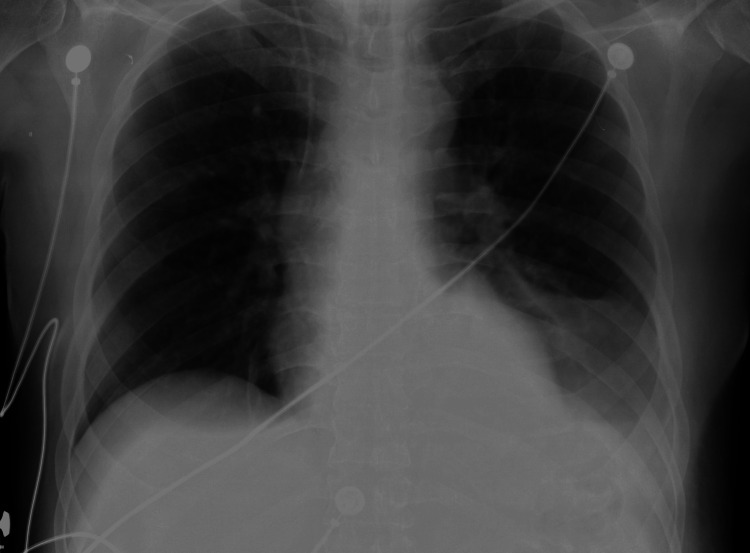
Pre-central venous catheterization (CVC) insertion chest X-ray

In July 2024, a 57-year-old male with no known comorbidity presented to the emergency department with a complaint of an altered level of consciousness and left-sided paralysis. Observations were taken, as mentioned in Table 1, and an examination was done. Initial management was given as per emergency protocol on suspicion of ischemic injury or stroke, including IV (intravenous) normal saline 100 cc/hour and high-flow oxygen, and supportive symptomatic medications were administered. Baseline workup was done (Table 2), and non-contrast CT was advised. A non-contrast CT scan of the head revealed an infarct in the right middle cerebral artery territory (Figure 1), and a chest X-ray was done pre-operatively (Figure 2). Anesthesia fitness was taken. Decompression surgery was planned. With written and informed consent, the patient was prepared for surgery.

**Table 1:** Observation taken in the emergency department on arrival BP: blood pressure, HR: heart rate, T: temperature, SpO₂: peripheral capillary oxygen saturation, RR: respiratory rate, GCS: Glasgow Coma Scale, BSR: blood sugar random, mmHg: millimeters of mercury, mg/dL: milligrams per deciliter, bpm: beats per minute, Afebrile: no fever present

Vital sign	Value	Unit	Normal range	Interpretation
Blood pressure (BP)	100/70	mmHg	90/60–120/80	Low-normal; monitor perfusion
Pulse (heart rate)	112	Beats per minute (bpm)	60–100	Mild tachycardia
Temperature (T)	Afebrile	°C	36.0–37.5 °C	Normal
Oxygen saturation	96% (room air)	%	>94%	Normal
Respiratory rate (RR)	21	Breaths per minute	12–20	Slightly elevated
Glasgow Coma Scale (GCS)	14/15	Score	15	Mildly altered mental status
Blood sugar (BSR)	128	mg/dL	70–140 (random)	Normal

**Table 2:** Patient’s emergency lab investigations and normal reference range Hb: hemoglobin, WBC: white blood cells, PLT: platelets, PT: prothrombin time, aPTT: activated partial thromboplastin time, INR: International Normalized Ratio, AST: aspartate aminotransferase, SGOT: serum glutamic-oxaloacetic transaminase, ALP: alkaline phosphatase, BUN: blood urea nitrogen, Cr: creatinine, Na⁺: sodium, K⁺: potassium, Cl⁻: chloride, pCO₂: partial pressure of carbon dioxide, HCO₃⁻: bicarbonate, CRP: C-reactive protein, PCT: procalcitonin, Trop-I: troponin-I

Lab test	Patient’s value	Normal value	Unit / notes
Hemoglobin	14	12–16 (female), 13–17 (male)	g/dL
White blood cells (WBC)	9800	4,000–11,000	cells/µL
Platelets	28300	150,000–400,000	cells/µL
PT (prothrombin time)	11.3	11–13.5	seconds
aPTT (activated partial thromboplastin time)	27.2	25–35	seconds
INR (International Normalized Ratio)	0.11	0.8–1.2	Ratio
AST (SGOT)	24	10–40	U/L
ALP	56	44–147	U/L
Total bilirubin	0.6	0.3–1.2	mg/dL
Urea (BUN)	14	7–20	mg/dL
Creatinine	0.9	0.6–1.3	mg/dL
Sodium (Na⁺)	134	135–145	mmol/L
Potassium (K⁺)	3.7	3.5–5.0	mmol/L
Chloride (Cl⁻)	102	96–106	mmol/L
pH	7.38	7.35–7.45	No unit
pCO₂	33	35–45	mmHg
HCO₃⁻	24	22–26	mmol/L
CRP (C-reactive protein)	3	<5	mg/L
Procalcitonin (PCT)	<0.1	<0.1	ng/mL
Troponin-I (Trop-I)	<0.10	<0.04	ng/mL (varies by assay)
Serum albumin	3.8	3.5–5.0	g/dL

**Figure 3:**
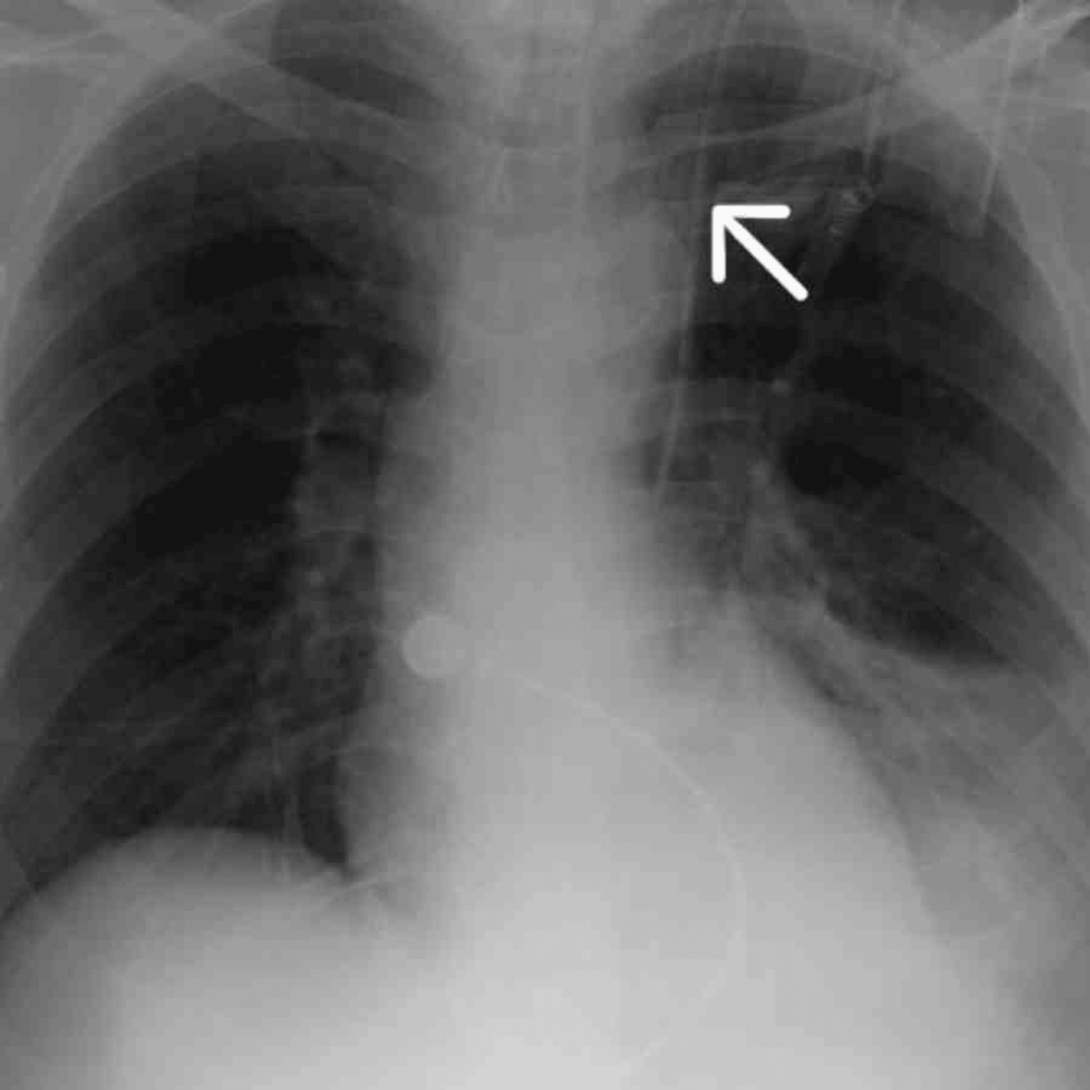
X-ray chest anteroposterior (AP) view The white arrow shows the catheter placement.

Prior to surgery, a central venous catheter (CVC) was inserted via the right internal jugular vein under ultrasound guidance to provide access for fluid resuscitation and vasopressor support during the procedure. There were no intraoperative or postoperative complications. Postoperatively, the patient was shifted to the ICU for initial vital and clinical stabilization under close monitoring as per ICU protocols. Later, the patient was transferred to the ward, where management included Glasgow Coma Scale (GCS) monitoring, input and output charting, head elevation at 30 degrees, intravenous fluids, antibiotics, antipyretics, antiemetics, mannitol, deep vein thrombosis (DVT) prophylaxis, and prophylactic antiepileptic drugs. The patient was allowed oral intake, passed urine spontaneously, and participated in postoperative mobilization, physiotherapy, and rehabilitation. Gradually, the patient showed clinical and vital signs showed improvement, although the clinical course necessitated an extended stay in the intensive care unit (ICU). After 10 days, routine replacement of the central line was performed. This time, the CVC was inserted through the left internal jugular vein. A chest X-ray was obtained to confirm appropriate catheter placement (Figure 3).

**Figure 4:**
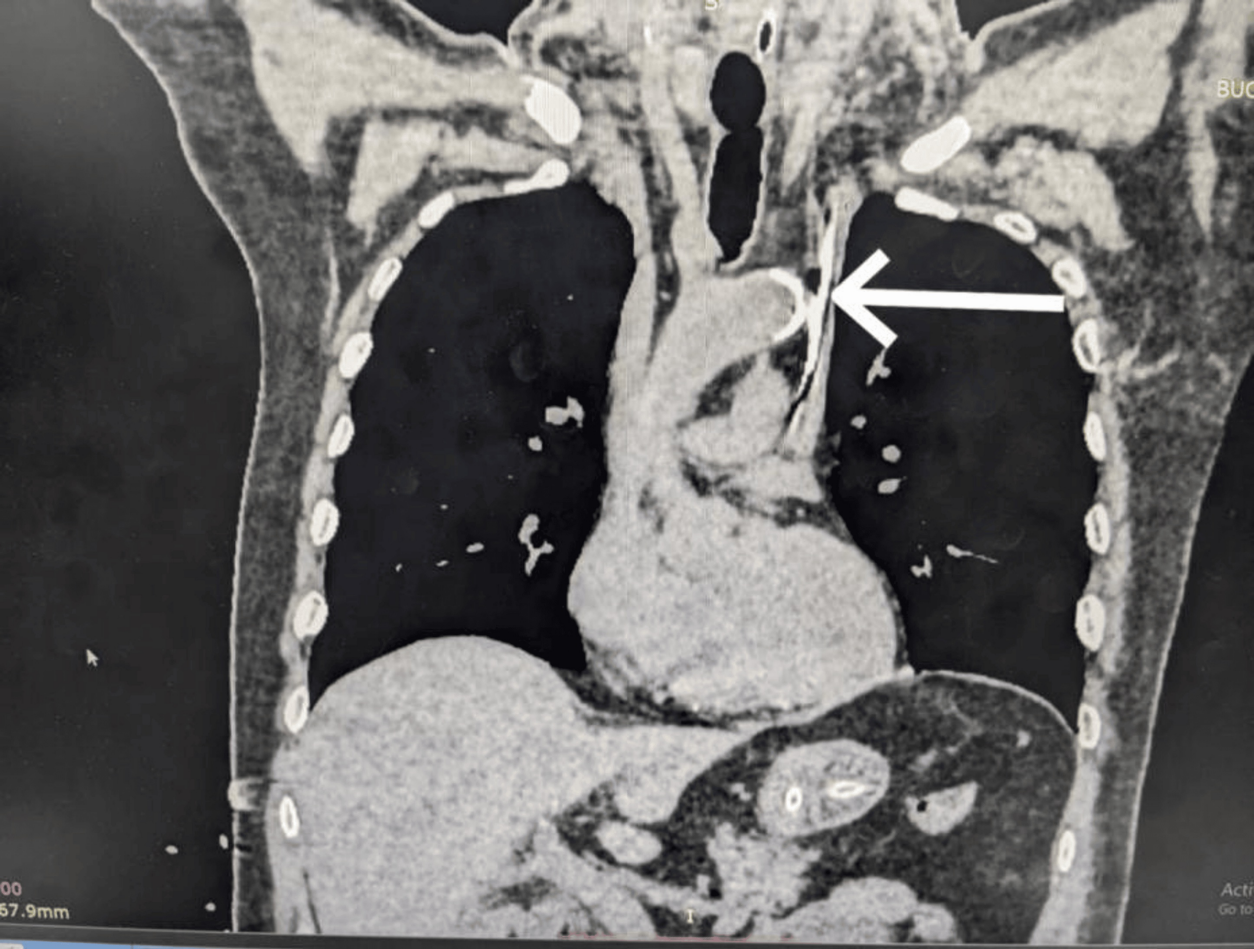
CT chest with the coronal view showing bilateral superior vena cava (SVC)

**Figure 5:**
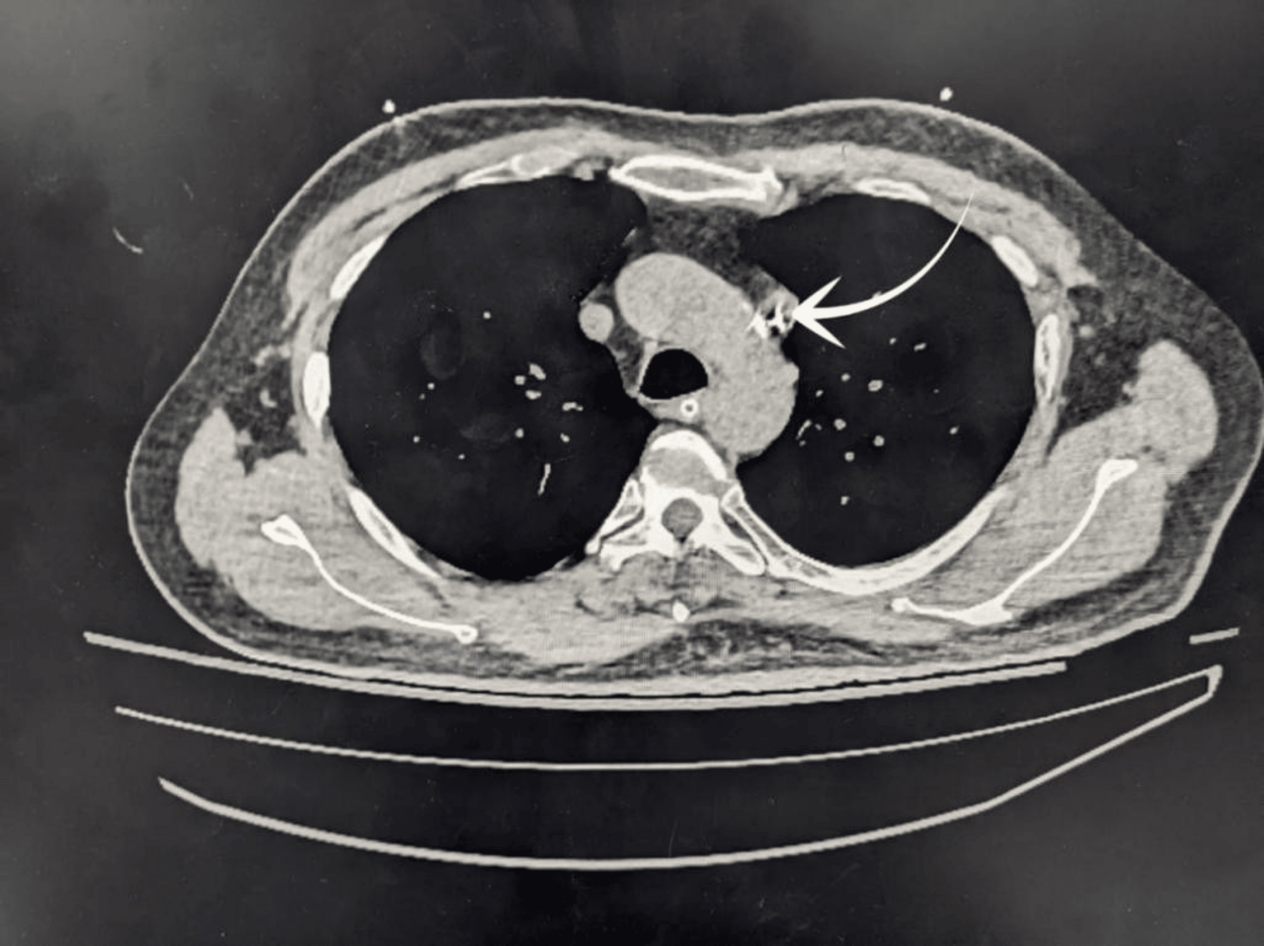
CT chest (axial view) showing bilateral superior vena cava (SVC) with central venous pressure (CVP) in the left SVC pointed by an arrow

Typically, a catheter inserted via the left internal jugular vein enters the left brachiocephalic vein, which crosses the midline anterior to the aortic arch to join the right-sided SVC and drain into the right atrium. However, in this case, the chest radiograph showed the catheter running along the left para-mediastinal border without crossing to the right side, which raised suspicion of an underlying vascular anomaly. A contrast-enhanced CT scan of the chest was performed, revealing the presence of a PLSVC draining into the coronary sinus (Figures 4, 5). Contrast-enhanced CT scan of the chest confirmed the coexistence of both left and right superior vena cava (bilateral SVC) with no other congenital abnormalities. The catheter functioned appropriately, and no complications were observed. The patient remained stable and continued to recover in the ICU. Later, the patient was discharged home on OPD (outpatient department) with oral medications and physiotherapy and was advised to follow-up.

## Discussion

Bilateral SVC is a relatively common vascular anomaly, particularly observed in pediatric patients undergoing surgical correction of congenital heart diseases [7]. While often asymptomatic, this anatomical variant can pose significant challenges during invasive procedures such as CVC, pacemaker placement, or cardiopulmonary bypass. As such, healthcare providers involved in perioperative care, interventional cardiology, and critical care should be familiar with this anomaly to avoid misinterpretation of imaging findings or complications during line placement.

One of the most frequent causes of bilateral SVC is PLSVC, which results from the embryologic failure of the left anterior cardinal vein to regress. PLSVC is classified into four distinct types based on their anatomical pathways and associated hemodynamic consequences [8,9]. The most common variant, type I, accounts for approximately 90% of all cases. In this type, PLSVC drains into the right atrium through an enlarged coronary sinus (CS), allowing venous return to follow the normal circulatory route. These patients typically do not have associated structural cardiac abnormalities [10], and a central venous catheter positioned through the PLSVC may remain in place without causing significant clinical issues.

Type II PLSVC is similar in that it also drains into the right atrium via the coronary sinus. However, this type features an additional open communication with the left atrium, resulting in a minor right-to-left shunt. Type III represents a more significant deviation, where the PLSVC bypasses the coronary sinus and drains directly into the left atrium, leading to unfiltered venous blood entering systemic circulation. Type IV is the rarest and involves coronary sinus atresia, with the PLSVC draining into the left atrium due to the absence or blockage of the coronary sinus. Both Type III and IV can lead to clinically relevant right-to-left shunting and subsequent systemic desaturation [11].

Patients with types II, III, or IV PLSVC often present with symptoms related to systemic hypoxemia, such as cyanosis, reduced exercise tolerance, dizziness, or fatigue. In more severe cases, they may experience syncope due to inadequate oxygen delivery. The mixing of deoxygenated venous blood with oxygenated arterial blood is responsible for these clinical features [12]. In such scenarios, placement of a central venous catheter through the PLSVC is discouraged due to the risk of systemic embolization, air embolism, or paradoxical embolism. If such a catheter is placed inadvertently, it should be removed promptly [13], and the patient should be referred for further evaluation and cardiothoracic consultation.

Surgical management of PLSVC depends on the patient’s unique anatomy and the hemodynamic significance of the anomaly. If a bridging vein, such as the innominate vein, is sufficiently large, the PLSVC can often be ligated without consequence. However, if the bridging vein is underdeveloped, absent, or if the right SVC is missing, ligation may compromise venous return. In such cases, surgical redirection of the PLSVC to the right atrium or coronary sinus is preferred to maintain physiologic circulation and eliminate abnormal shunting [14]. Early diagnosis and appropriate surgical planning are crucial in preventing complications such as chronic cyanosis or paradoxical embolism in these patients [15-17].

## Conclusions

A rare but clinically relevant vascular abnormality is bilateral SVC, which results from a PLSVC. Although it is usually asymptomatic, it can cause problems during thoracic surgery, cardiac device insertion, and CVC. To prevent operational problems, it is crucial to make an accurate diagnosis using imaging methods like echocardiography or CT angiography. Clinicians should be proficient in identifying and treating this variation. To ensure individualized care for patients with PLSVC, a special case profile should be created for them. Knowing about this anomaly facilitates improved planning for both elective and emergency interventions. A good grasp of anatomy helps minimize intraoperative dangers and avoid misinterpretations. Patient outcomes may be dramatically improved by early detection and knowledgeable clinical approaches. Familiarity with bilateral SVC ultimately aids in more effective and secure patient care.
